# Transdiagnostic subtyping of males with developmental disorders using cortical characteristics

**DOI:** 10.1016/j.nicl.2020.102288

**Published:** 2020-05-26

**Authors:** Takashi Itahashi, Junya Fujino, Ryu-ichiro Hashimoto, Yoshiyuki Tachibana, Taku Sato, Haruhisa Ohta, Motoaki Nakamura, Nobumasa Kato, Simon B. Eickhoff, Samuele Cortese, Yuta Y. Aoki

**Affiliations:** a Medical Institute of Developmental Disabilities Research, Showa University, Tokyo, Japan; b Department of Language Sciences, Graduate School of Humanities, Tokyo Metropolitan University, Tokyo, Japan; c Division of Infant and Toddler Mental Health, Department of Psychosocial Medicine, National Center for Child Health and Development, Tokyo, Japan; d Institute of Systems Neuroscience, Medical Faculty, Heinrich Heine University Düsseldorf, Düsseldorf, Germany; e Institute of Neuroscience and Medicine, Brain & Behaviour (INM-7), Research Centre Jülich, Jülich, Germany; f New York University Child Study Center, New York, NY, USA; g Center for Innovation in Mental Health, Academic Unit of Psychology, University of Southampton, UK; h Clinical and Experimental Sciences (CNS and Psychiatry), Faculty of Medicine, University of Southampton, UK; i Solent NHS Trust, Southampton, UK; j Division of Psychiatry and Applied Psychology, School of Medicine, University of Nottingham, Nottingham, UK

**Keywords:** Attention-deficit/hyperactivity disorder, Autism spectrum disorder, Cortical thickness, HYDRA, Subtype

## Abstract

**Background:**

Autism spectrum disorder (ASD) and attention-deficit/hyperactivity disorder (ADHD) are biologically heterogeneous and often co-occur. As within-diagnosis heterogeneity and overlapping diagnoses are challenging for researchers and clinicians, identifying biologically homogenous subgroups, independent of diagnosis, is an urgent need.

**Methods:**

MRI data from 148 adult males with developmental disorders (99 primary ASD, mean age = 31.7 ± 8.0, 49 primary ADHD; mean age = 31.7 ± 9.6) and 105 neurotypical controls (NTC; mean age = 30.6 ± 6.8) were analyzed. We extracted mean cortical thickness (CT) and surface area (SA) values using a functional atlas. Then, we conducted HeterogeneitY through DiscRiminant Analysis (HYDRA) to transdiagnostically cluster and classify individuals. Differences in diagnostic likelihood and clinical symptoms between subtypes were tested. Sensitivity analyses tested the stability of the number of subtypes and their membership by excluding 13 participants diagnosed with both ASD and ADHD and by using a different atlas.

**Results:**

In relation to both CT and SA, HYDRA identified two subtypes. The likelihood of ASD or ADHD was not significantly different from the chance of belonging to any of these two subtypes. Clinical characteristics did not differ between subtypes in either CT or SA based analyses. The high consistency in membership was replicated when utilizing a different atlas or excluding people with dual diagnoses in CT (dice coefficients > 0.94) and in SA (>0.88).

**Conclusion:**

Although the brain-derived subtypes do not match diagnostic groups, individuals with developmental disorders were successfully and stably subtyped using either CT or SA.

## Highlights

• Overlapping diagnosis and within-diagnosis heterogeneity was often reported in ASD.
• ASD and ADHD were subtyped regardless of diagnosis using cortical characteristics.
• The analysis revealed the number of subtypes as two.
• The boundary of the subtypes did not match the diagnostic boundary.
• The membership of subtypes was robust against the choice of an atlas.

## 1 Introduction

Autism spectrum disorder (ASD) is a developmental disorder characterized by two core symptoms: social communication impairment and restricted interest and repetitive behavior (American Psychiatric Association, 2013). The prevalence of ASD is estimated to be more than 1% (Xu et al., 2018); symptoms are also observed in those without the full diagnosis (Hoekstra et al., 2007). Attention-deficit/hyperactivity disorder (ADHD) is another developmental disorder characterized by age-inappropriate inattentive and/or hyperactive-impulsive symptoms (American Psychiatric Association, 2013). Similarly, ADHD symptoms are observed also in people without the full diagnosis (Lubke et al., 2009). Biological findings, such as genetic factors contributing to the symptoms and traits, familial aggregation, and endophenotype, transcend the diagnostic boundary in both ASD and ADHD (Chen et al., 2017, Clarke et al., 2016, Gau and Shang, 2010, Sandin et al., 2014, Stergiakouli et al., 2015, Yamagata et al., 2018). Additionally, ASD and ADHD symptoms also transcend the boundary between these two disorders. Indeed, one-third of individuals with ASD meet ADHD diagnosis and vice versa and even more show traits of the other disorder (Grzadzinski et al., 2011, Grzadzinski et al., 2016, Reiersen et al., 2008). Co-occurrence happens not only in core but also in non-core symptoms, such as hyper- and hypo-sensitivity (Little et al., 2018, Ohta et al., 2019, Pan et al., 2009). Besides the overlap in these clinical symptoms, ASD and ADHD show familial co-aggregation (Ghirardi et al., 2018, Jokiranta-Olkoniemi et al., 2016), suggesting their shared genetic etiology (Stergiakouli et al., 2017). Although results vary, neuroimaging studies demonstrated some similarities in atypical cortical structure, white matter, and function (Ameis et al., 2016, Brieber et al., 2007, Chantiluke et al., 2014, Christakou et al., 2013). On top of such shared atypicality, neural bases of ASD symptoms in individuals with ADHD are similar to those in individuals with ASD (Baribeau et al., 2019). These overlapping features contribute, at least in part, to the within-diagnosis heterogeneity and inconsistency in findings from studies contrasting diagnostic groups (Insel, 2014). Identifying highly biologically homogeneous subtypes, independent of the clinical diagnosis, is an urgent need in the research field of developmental disorders.

In this context, previous functional MRI studies focused on features that ensure phenotypic homogeneity in ASD, in ADHD, and in both (Kernbach et al., 2018, Lin et al., 2018, Tang et al., 2019). However, findings are mixed. While a study including individuals with ADHD highly homogeneous in terms of demographic characteristics showed high homogeneity in neural characteristics within the diagnostic boundary (Lin et al., 2018), another study with a larger sample size showed neural heterogeneity within ASD diagnosis (Tang et al., 2019). Of note, a study with both ASD and ADHD detected brain transdiagnostic features that map clinical characteristics independent of diagnoses (Kernbach et al., 2018). Two studies attempted to subtype clinical participants to address the unstable numbers of subtypes (Kernbach et al., 2018, Lin et al., 2018). One possible reason for instability is too many degrees of feature utilized to subtype. Indeed, two studies relying on structural MRI, which has a smaller number of degrees of feature than fMRI, used different clustering methods aimed to subtype individuals with ASD reported the optimal number of subtypes as three (Chen et al., 2019, Hong et al., 2018). Another possible reason is that previous clustering methods relied on the similarity of fMRI signal among subjects and can be vulnerable to cofounding signal related to non-pathophysiological characteristics. Thus, they might have reflected normal inter-subject variability, rather than highlighting the heterogeneity.

To address these issues from previous studies, we recruited adult males with ASD and those with ADHD as well as adult male neurotypical controls (NTC) and applied Heterogeneity through Discriminative Analysis (HYDRA) to structural MRI data (Varol et al., 2017). HYDRA is a hybrid method where clustering and classification are conducted simultaneously. A standard classification approach (e.g., support vector machine) attempts to identify the pathophysiological features spanning the clinical group from the NTC group by drawing a decision boundary between the groups without considering the heterogeneity within the clinical group. In contrast, HYDRA attempts to find subtypes within the clinical group while maximizing margin boundaries between the clinical and NTC groups. This unique ability allows us to identify biologically homogeneous subtypes, each of which preserve different aspects of pathophysiological features of developmental disorders, i.e., differences to typically developing controls, rather than picking up features which have a non-pathophysiological origin, such as head motion, bed vibration, or instability of magnetic field strength. As they reflect different aspects of the cortex, we utilized two different cortical parameters: cortical thickness (CT) and surface area (SA) with high test–retest reliability (Iscan et al., 2015).

## 2 Materials and methods

### 2.1 Participants

MRI data of 148 adult males with a developmental disorder(s) (99 primary ASD diagnosis and 49 primary ADHD diagnosis) as well as 105 NTC participants were selected from a larger sample. Thirteen individuals had both diagnoses. Because of the nature of the current study aimed to transdiagnostically subtype people, we gave priority to people with a single diagnosis to include. Thus, the number of people with dual diagnoses is smaller than the estimated. The diagnostic process of the current study is detailed elsewhere (Ohta et al., 2019). Briefly, a multidisciplinary team, including psychiatrists and psychologists, reviewed developmental history and made clinical diagnoses according to the DSM-IV-TR criteria. A diagnosis of ASD was further supported by the Autism Diagnostic Observation Schedule-1 or -2 (ADOS) (*n* = 74) (Gotham et al., 2009, Gotham et al., 2007). Five individuals did not satisfy the diagnostic criteria of ASD on the ADOS (four had a total score of 6; one had a total score of 7 but the communication score was 1). In these cases, at least two psychiatrists carefully reviewed the charts and confirmed the clinical diagnosis. ASD and ADHD traits were evaluated using the Autism Spectrum Quotient and Conners’ Adult ADHD Rating Scales, respectively (Baron-Cohen et al., 2001, Conners et al., 1999). The intelligence quotient (IQ) of participants in clinical groups was estimated using either the Wechsler Adult Intelligence Scale-Third Edition (WAIS-III) or WAIS-Revised (WAIS-R) (Wechsler, 1997, Wechsler and De Lemos, 1981). Seventy-two participants were taking medication at the time of scan (36 ASD and 36 ADHD). NTC participants were recruited via an advertisement or through an acquaintance. A lack of any psychiatric diagnosis in the NTC group was confirmed using the Mini-International Neuropsychiatric Interview (Sheehan et al., 1998). Full-scale IQ in the NTC group was estimated using a Japanese version of the National Adult Reading Test (Matsuoka et al., 2006). Exclusion criteria for all the participants included taking antipsychotics, any history of head trauma, any serious medical condition, substance abuse, or IQ below 80.

### 2.2 MRI data acquisition

All MRI data were acquired using a 3.0 T MRI scanner (MAGNETON Verio, Siemens Medical Systems, Erlangen, Germany) with a 12-channel head coil. T1-weighted images were acquired with an MPRAGE sequence (TR: 2.3 s, TE: 2.98 ms, flip angle: 9°, FOV: 256 mm, matrix size: 256 × 256, slice thickness: 1 mm, 240 sagittal slices, voxel size: 1 × 1 × 1 mm).

### 2.3 Structural MRI data preprocessing

Structural MRI data was processed using FreeSurfer version 6.0.1 (Dale et al., 1999, Fischl et al., 1999). This algorithm performs a series of preprocessing steps, including spatial normalization, bias field correction, intensity normalization, skull-stripping, segmentation, and reconstruction of surface mesh. We computed the aforementioned two parameters: CT and SA. For each parameter, we, then, extracted the mean values using the Schäfer's 400 cortical atlas that parcellated cortex into functionally homogeneous parcels using intrinsic functional connectivity (Schaefer et al., 2018). To test the robustness of findings against atlas choice, we also computed the mean values using Destrieux's cortical atlas (Destrieux et al., 2010). At the same time, utilizing a different atlas challenges the stability of the findings against the degree of features as the Destrieux's cortical atlas consists of 148 regions of interest (ROIs) while the Schaefer's 400 cortical atlas consists of 400 ROIs.

### 2.4 Number of subtypes

We used a recently developed semi-supervised learning method, referred to as HYDRA, the code of which is publicly available (https://github.com/evarol/HYDRA) (Varol et al., 2017). Using the cortical parameter (i.e., CT or SA) of 400 ROIs while including age and handedness as nuisance covariates, we defined neuroanatomical subtypes, regardless of clinical diagnoses. This method is based on the classification framework, in which it compares participants with developmental disorders and NTCs to identify *k* clusters within the group of participants with developmental disorders in a data-driven manner. We set the hyperparameters, such as the number of iterations, that of consensus clustering steps, and regularization parameter, based on a previous study (Varol et al., 2017). Consistent with previous studies with this method, we set multiple clustering solutions from two to 10 clusters, to obtain a range of possible solutions. The adjusted rand index (ARI) was computed using 10-fold cross-validation to assess the stability of each clustering solution. In this study, we considered a solution with the highest ARI value as the optimal number of clusters (Steinley, 2004). Once the optimal number of clusters was determined, a permutation test with 5000 iterations was then performed to examine its statistical significance. At each iteration, labels were shuffled. HYDRA was then applied, and the ARI for the shuffled data was computed to construct the null distribution. The actual ARI was then compared to the null distribution to examine its statistical significance. The threshold for statistical significance was set to *P* < 0.05. To further confirm the stability of clustering solutions, we computed two additional metrics, the Hamming distance and the Rand index, representing dissimilarity and similarity, respectively, between different clustering solutions. Thus, lower Hamming distance and higher Rand index indicate more stable clustering solutions.

### 2.5 Statistical analyses

#### 2.5.1 Clinical characteristics of each subtype

##### 2.5.1.1 Two-group comparison

Once subtypes were identified, two-tailed, two-sample tests were performed, to investigate differences in clinical characteristics, such as age, handedness, IQ, AQ, and CAARS, among subtypes as well as differences between subtype and the NTC group. The threshold of statistical significance was set at *P* < 0.05 after false discovery rate (FDR) correction. Chi-square tests examined whether the likelihood of the diagnosis of ASD or ADHD is statistically significantly different from the chance.

##### 2.5.1.2 Three-group comparison

One-way analysis of variance (ANOVA) was performed to examine the effects of primary diagnoses on demographic characteristics. The threshold of statistical significance was set at *P* < 0.05 for demographic characteristics, while those for cortical parameters were set at *P* < 0.05 after FDR correction.

### 2.6 Cortical characteristics of subtypes

We conducted two-tailed, two-sample tests to examine differences in cortical parameters among subtypes as well as differences between subtype and the NTC group. The threshold of statistical significance was set at *P* < 0.05 after false discovery rate (FDR) correction. The analyses were conducted for CT and SA, separately.

### 2.7 A convergence of area with differences

Once statistically significant differences were observed between subtypes and the NTC group, conjunction analyses were performed to identify brain regions consistently exhibiting alterations across conditions. To increase the interpretability of results, we also calculate the percentage of altered brain regions in each of seven resting-state networks (Yeo et al., 2011).

### 2.8 Membership consistency

To evaluate membership consistency across four HYDRA (2 atlases × 2 inclusion criteria), we calculate dice coefficients while considering the subtypes obtained using the functional atlas including individuals with dual diagnoses as a reference. These analyses were performed in CT and SA, separately.

### 2.9 Conventional comparison between diagnostic groups

#### 2.9.1 Demographic characteristics

Age, handedness, IQ, AQ, and CAARS scores were compared across three diagnostic groups (ASD, ADHD, and NTC) using ANOVA. Statistical threshold was set at *P* < 0.05 with FDR correction. Turkey’s post-hoc analyses were conducted when ANOVA showed statistical significance.

### 2.10 Cortical parameters

In HYDRA, we adopted a whole-brain approach instead of focusing on ROI. To examine regional differences in cortical parameters between diagnostic groups, we conducted a conventional ANOVA. We utilized the functional atlas and set statistical significance at *P* < 0.05 after FDR correction. All analyses were conducted in Matlab.

## 3 Results

### 3.1 Number of subtypes

With 10-fold cross-validation, HYDRA using CT identified that two subtypes (i.e., *k* = 2) showed the highest ARI (ARI = 0.85 ± 0.02 [mean ± standard deviation]) and the highest statistical significance (*p* < 0.001), indicating that the individuals with developmental disorders were clustered into two subtypes (CT-subtype1 and CT-subtype2). The number of clusters remained two when we conducted HYDRA with SA (ARI = 0.75 ± 0.02, *p* < 0.001) (SA-subtype1 and SA-subtype2). The optimal numbers of subtypes were unchanged even when excluding 13 people with a dual diagnosis and/or using the anatomical atlas (Supplementary Fig. 1). Furthermore, the optimal numbers of subtypes was corroborated by the supplementary exploration of two additional metrics, i.e., the Hamming distance (CT: 0.12 ± 0.03, SA: 0.08 ± 0.03) or Rand index (CT: 0.89 ± 0.02, SA: 0.92 ± 0.03; Supplementary Fig. 2) to assess cluster stability.

### 3.2 Clinical characteristics of each subtype

Forty-nine individuals with ASD and 25 people with ADHD were assigned to CT-subtype1, while CT-subtype2 included 50 individuals with ASD and 24 individuals with ADHD (Supplementary Table 1). Chi-square test showed that the likelihood of a diagnosis of ASD or ADHD did not statistically significantly different from the chance. There was no significant difference in clinical symptoms between these two subtypes. Similarly, the statistical differences in the diagnosis likelihood or clinical symptoms severity were not observed even when excluding individuals with dual diagnoses and/or using the anatomical atlas (Supplementary Table 1). The results with SA were substantially similar to CT (Supplementary Table 2).

### 3.3 Cortical characteristics of subtypes

#### 3.3.1 CT

**Fig. 1:**
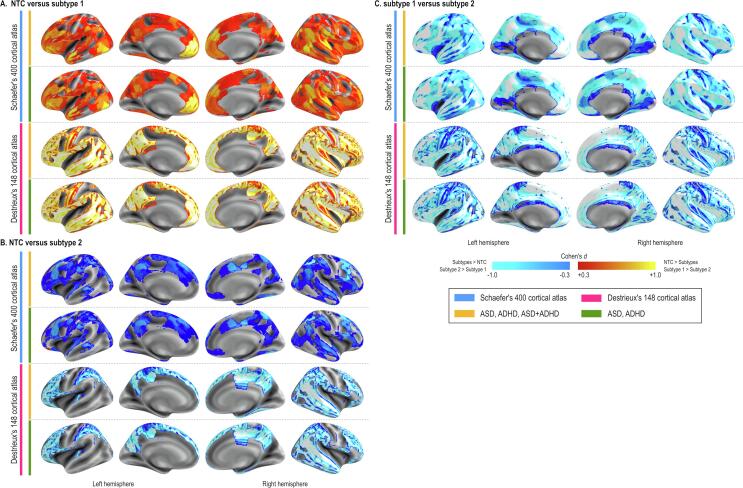
Results of cortical thickness subtyping. The difference in cortical thickness (CT) between neurotypical controls (NTC) and CT-subtype1 is shown (A). The difference was diffusely distributed with little exception, such as the primary vision area, dorsal anterior cingulate cortex. Compared with the NTC, CT-subtype2 also showed diffusely low CT values with the largest difference in the dorsolateral prefrontal cortex. The area with the significand difference in CT values between CT-subtype1 and CT-subtype2 covers almost the entire brain. The threshold of statistical significance was set at *P* < 0.05 after FDR correction.

The independent t-tests showed that compared with NTC individuals who were assigned to CT-subtype1 had high CT values in diffuse brain regions (Fig. 1A 1st row). This pattern did not change in the sensitivity analysis excluding people with dual diagnoses (Fig. 1A 2nd row), as well as utilizing the anatomical atlas to extract CT values (Fig. 1A 3rd and 4th rows).

#### 3.3.2 SA

**Fig. 2:**
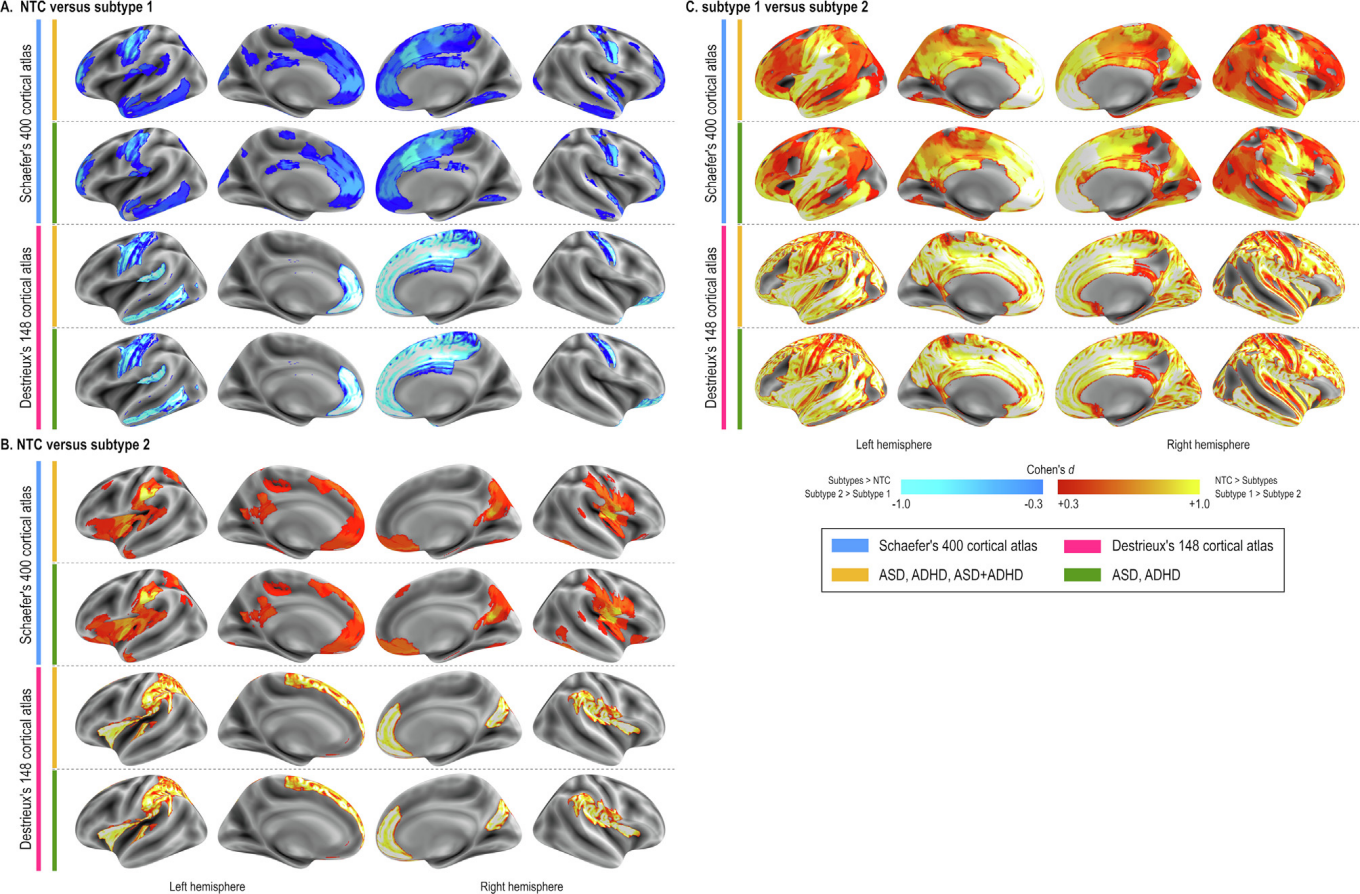
Results of surface area subtyping. The difference in surface area (SA) between neurotypical controls (NTC) and SA-subtype1 is shown (A). The similar pattern was observed when we utilized the functional atlas with (the first raw) or without individuals with dual diagnoses (the second row) and when we adopted the anatomical atlas (the third and fourth rows). Sub-panel B shows the results of comparison between NTC against SA-subtype2, while subpanel C shows the difference in SA values between SA-subtype1 and SA-subtype2. The threshold of statistical significance was set at *P* < 0.05 after FDR correction.

The analysis with SA showed relatively localized differences between subtypes and NTC. The SA-subtype1 showed a large area of SA difference in the medial region while less was observed in the lateral region with the largest difference in the primary motor and sensory areas (Fig. 2A 1st row). SA-subtype2 showed even less area of SA difference in the medial part. On the other hand, the posterior insula showed a large SA value difference between SA-subtype2 and NTC (Fig. 2B 1st row). This pattern did not change with other sub-analyses (Fig. 2A.B 2nd-4th rows). SA-subtype1 and SA-subtype2 showed diffuse areas with differences in SA values (Fig. 2C).

### 3.4 A convergence of area with differences

**Fig. 3:**
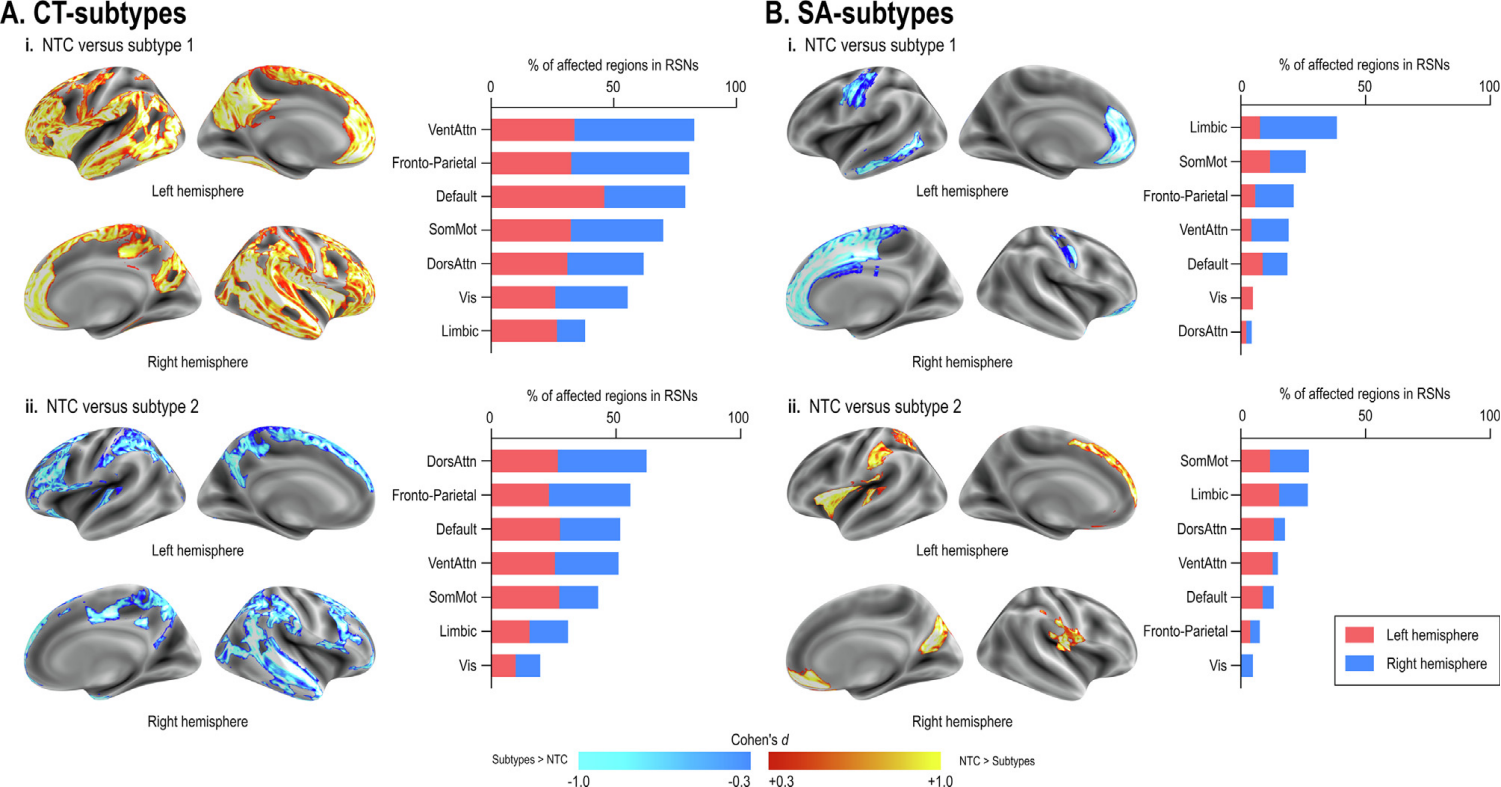
Convergence of area across subtypes. Although we defined the results of HYDRA using the functional atlas with the whole participants, we conducted other three analyses utilizing the anatomical atlas (2 atlases) and with/without people with dual diagnoses (2 inclusion criteria for individuals). The subpanels (A) show the convergence of the results across the subtypes (left panel) and the percentage of area affected in each resting state network (right panel). We performed the same analyses adopted SA. The convergence of the area affected across subtypes (2 atlases × 2 participants inclusion criteria) was shown in the subpanel (B).

CT-subtypes exhibited diffuse albeit an inverse pattern of alterations when compared to the NTC group. Both subtypes exhibited alterations mainly in the dorsal attention, default mode, fronto-parietal, and ventral attention networks (Fig. 3A). On the other hand, SA-subtypes exhibited relatively localized alterations. SA-subtype1 exhibited right-dominant alterations mainly in the limbic network, while SA-subtype2 showed alterations in the somatomotor and limbic networks (Fig. 3B).

### 3.5 Membership consistency

#### 3.5.1 CT

Compared with the primary CT results (HYDRA using the functional atlas including those with dual diagnoses), the HYDRA using the functional atlas excluding those with dual diagnoses showed the dice coefficient of 0.96. On the other hand, HYDRA using the anatomical atlas showed high consistency with the primary CT results with (0.94) and without (0.95) people with dual diagnoses.

#### 3.5.2 SA

Compared with the primary SA results, the functional atlas analysis excluding people with dual diagnoses (0.99) and the anatomical atlas analyses with (0.90) and without (0.88) individuals with dual diagnoses revealed high consistency in membership.

### 3.6 Conventional comparison between diagnostic groups

#### 3.6.1 Demographic characteristics

There were no statistically significant differences in age, handedness, or IQ across three diagnostic groups (ASD, ADHD, and NTC) (see Supplementary Table 3). With a few exceptions in subscales, AQ scores were higher in individuals with ASD compared with individuals with ADHD, whose AQ scores were higher than TD. Likewise, CAARS scores were generally higher in individuals with ADHD compared with people with ASD, and individuals with ASD had higher CAARS scores compared with NTC.

#### 3.6.2 Cortical parameters

**Fig. 4:**
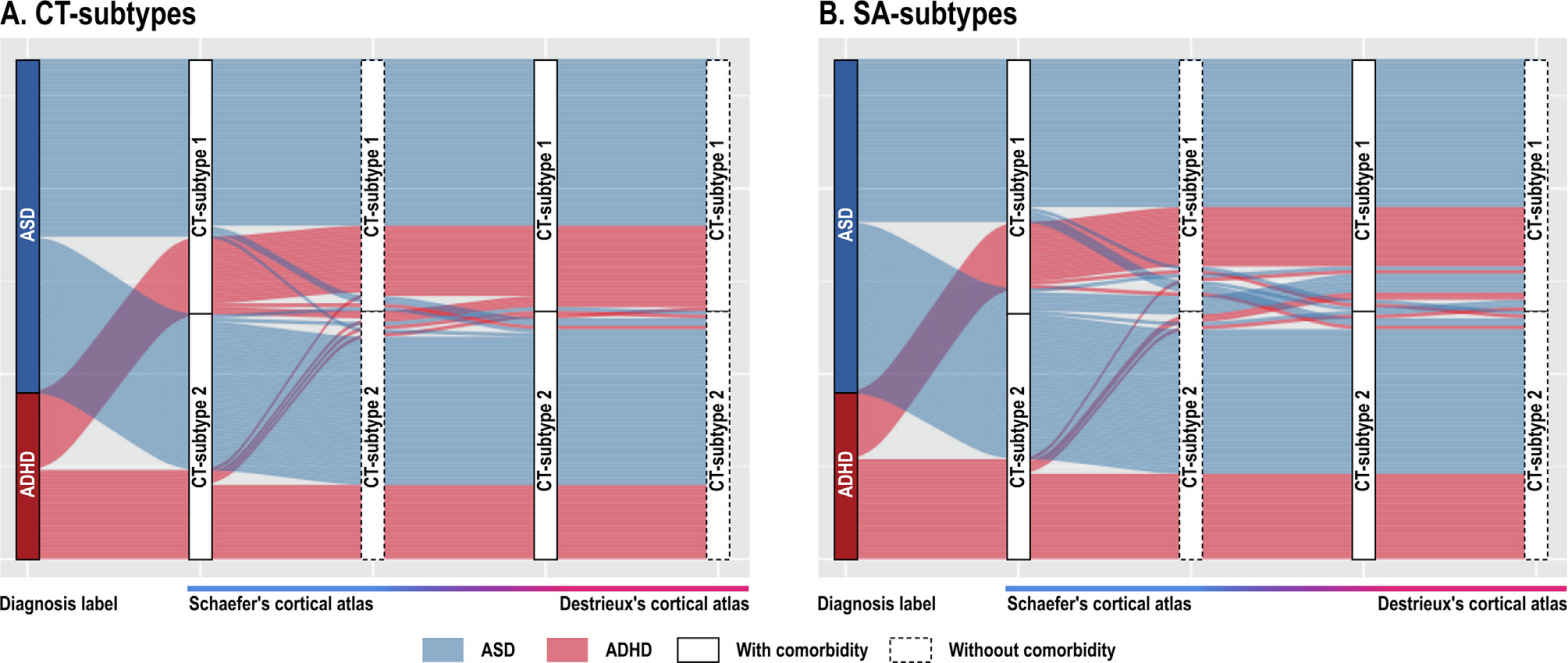
Sanky diagram across the diagnoses and subtypes. Cortical thickness (CT) The current study enrolled 99 individuals with autism spectrum disorder (ASD) and 49 individuals with attention-deficit/hyperactivity disorder (ADHD). Fifty-two individuals with ASD and 23 individuals with ADHD were assigned to the CT-subtype1, while 47 individuals with ASD and 26 individuals with ADHD were assigned to CT-subtype2, when we run the analysis using CT values extracted from the functional atlas (the left column). Further, we conducted the analyses excluding people with dual diagnoses and utilizing an anatomical atlas instead of the functional atlas. Across these four analyses, 45 individuals with ASD and 18 individuals with ADHD were consistently assigned to the subtype1, while 40 people with ASD and 20 people with ADHD were consistently assigned to the subtype2. Surface area (SA) In the SA analysis using a functional atlas, 49 individuals with ASD and 20 individuals with ADHD were assigned to SA-subtype1, while 50 individuals with ASD and 29 individuals with ADHD were assigned to SA-subtype2. Across the four analyses, 40 individuals with ASD and 16 people with ADHD were consistently assigned to the subtype1. On the other hand, 39 individuals with ASD and 23 individuals with ADHD were consistently assigned to the subtype2.

ANOVA showed that there were no statistically significant group differences in CT or SA across diagnostic groups (Fig. 4).

## 4 Discussion

Using a novel approach, we aimed to transdiagnostically subtype, based on structural MRI features, individuals with developmental disorders to reach high homogeneity in cortical characteristics. As we expected, the number of subtypes was stable in both CT and SA. Further, the membership assignment was robust against the atlas (functional vs. anatomical) and exclusion of individuals with dual diagnoses (ASD + ADHD). In addition, the biologically homogeneous subtypes did not match clinical diagnoses. However, in contrast to our expectation, biologically homogeneous subtypes did not show any phenotypic homogeneity.

The current study showed the same number of subtypes as the diagnoses (ASD and ADHD). Further, the analyses showed the stability of the membership against differences in atlas and participants. In general, the larger number of features resulted in overfitting and the greater number of subtypes, while a relatively smaller number of features exhibited stability in the clustering results. Given that structural MRI data has fewer features due to the lack of temporal resolution compared with fMRI, utilizing structural MRI may explain the stability of the results. Besides, the robustness against the number of features in structural data was shown by demonstrating the same number of subtypes using two different atlases (148 vs. 400 ROIs). At the same time, although a number of studies have reported atypical brain structure in individuals with ASD and those with ADHD (Anagnostou and Taylor, 2011, Vaidya, 2012), using structural data might have led to the negative findings that subtypes do not match clinical diagnoses. There are some potential reasons for the negative findings. First, a number of hypotheses on atypical functional connectivity were launched as potential pathophysiology in developmental disorder. For example, dysconnectivity within default mode network, atypical large-scale brain network, and imbalance between networks were considered in individuals with ADHD (Castellanos and Proal, 2012, Konrad and Eickhoff, 2010, Qian et al., 2019). In terms of ASD, atypical connectivity is reported even more often than in ADHD. For instance, a long-distance hypo- and short-distance hyper-connectivity hypothesis is still one of the compelling pathophysiology (Kana et al., 2014). Further, a number of fMRI studies reported atypical network parameters in ASD (e.g. (Wilkes and Lewis, 2018)). Second, in addition to functional connectivity, atypical structural connectivity is often reported in both ASD and ADHD (Uddin et al., 2017). However, as the cortical parameters did not address structural nor functional connectivity, they may not be the best way to examine pathophysiology of developmental disorders. It should be noted that the current findings cannot be generalized to fMRI nor diffusion tensor imaging. Future research with the multimodal data is expected to address this concern.

Although subtypes showed a diffuse difference, convergence analyses showed that brain regions in the ventral attention, fronto-parietal, and default mode networks were most affected in the CT subtyping. Albeit different modalities, the current findings were, at least in part, consistent with the findings of prior studies that attempted to subtype people with developmental disorders with a data-driven approach. Concretely, they demonstrated that brain-derived subtypes were characterized by functional connectivity involving default mode network in both transdiagnostic (Kernbach et al., 2018) and within diagnostic approaches (Tang et al., 2019). Intriguingly, Subtype1 of both CT and SA showed atypical values in the medial prefrontal cortex where prior studies contrasting individuals with ASD and NTC consistently showed atypicality with different modalities (Aoki et al., 2012, Carlisi et al., 2017). Given that *meta*-analyses of studies contrasting people with ADHD against NTC also showed atypicality in the brain region (Aoki et al., 2013, Norman et al., 2016), these results might have represented either the categorical effect of the diagnosis or the subclinical traits of the other disorder. A future study that disentangles the effects of both traits and diagnoses is expected.

There are some more possible reasons for the current findings showing that biologically homogeneous subtypes do not show homogeneity in phenotype, which is not consistent with one prior study that showed significant performance to classify ASD from ADHD using structural MRI data (Lim et al., 2013). First, although we deem that our data collected, evaluated, and diagnosed in one-site was not influenced by multiple confounding factors, such as a difference in a scanner, scan parameters, and ethnicity, the sample size is relatively small compared with open data sharing (Consortium, 2012, Di Martino et al., 2014b). Further, even though all the participants experienced both diagnostic evaluation and MRI scan, not all of them finished a psychological evaluation, such as CAARS an AQ. Thus, the statistical power to detect phenotypical differences became even smaller, which may contribute to the lack of difference in clinical measures. Second, to reduce bias, we adopted the whole-brain approach, which may underestimate the localized difference between diagnoses (Bethlehem et al., 2018, Uddin et al., 2017). Clustering analysis might have shown better performance if we focused on only a part of the brain where the atypical structure was frequently reported for ASD or ADHD (Samea et al., 2019, Yamagata et al., 2019). However, it should be noted that the conventional comparison between diagnostic groups in the current study did not show any difference in CT or SA, which made it unreasonable to focus on a part of the brain in the current participants. Third, although different trajectory patterns are expected for ASD and ADHD especially during childhood and adolescence (Di Martino et al., 2014a, Rommelse et al., 2017, Shaw et al., 2007, Wallace et al., 2010), the current study included only adults. Namely, the current study might categorize people with different developmental trajectories into the same group. Indeed, many studies with ASD and ADHD, including the prior study with statistically significant diagnosis prediction performance (Lim et al., 2013), enrolled children or adolescents (Ameis et al., 2016, Aoki et al., 2017). Fourth, because of practical reasons, some of the participants were not medication naïve. Although there was no significant difference in the likelihood of being under medication between two subtypes in any condition, the current findings may be biased by the medication (Nakao et al., 2011). However, it was not possible to run the analyses after excluding people under medicated, as the number of individuals with ADHD without medication was only 13.

Finally, the current findings may be interpreted that the clinical diagnoses do not match biological homogeneity. Atypical findings shared by ASD and ADHD were reported not only structural MRI but also other MRI modalities (Ameis et al., 2016, Bethlehem et al., 2017, Di Martino et al., 2013). Accumulation of evidence that showed overlapping abnormality across diagnoses calls for a next step: from contrasting clinical diagnostic groups to classification with biologically high homogeneity (Insel et al., 2010).

In conclusion, we classified people with developmental disorders based on either CT or SA. The likelihood of ASD or ADHD differed from chance in neither CT or SA based analyses. The current results suggest that transdiagnostic subtypes with high homogeneity in the cortical parameter do not match diagnostic groups. On the other hand, we demonstrated the stability of memberships and number of subtypes while clustering people with developmental disorders, indicating the feasibility of subtyping with high biological homogeneity. The results call for a paradigm shift from examining the biological difference between the diagnostic group to define maximally homogeneous transdiagnostic subtypes from both phenotypical and biological perspectives.

## Declaration of interest

SC: honoraria for talks on ADHD from: Association form Child and Adolescent Mental Health (CADDRA), British Association of Psychopharmacology (BAP), Canadian ADHD Alliance Resource (CADDRA), and Healthcare Convention.

All the other authors declare no conflict of interest.
