# Supplementary material for: Transdiagnostic subtyping of males with developmental disorders using cortical characteristics
Source: Neuroimage Clin. 2020 May 26;27:102288. doi: 10.1016/j.nicl.2020.102288 (PMC7284124; doi:10.1016/j.nicl.2020.102288)
Supplement: Supplementary Fig. 1 [file mmc1.docx]

**Supplementary Information**

**Title:**

Transdiagnostic subtyping of males with developmental disorders using cortical characteristics

**Authors:**

Takashi Itahashi,^a^ Junya Fujino,^a^ Ryu-ichiro Hashimoto,^a,b^ Yoshiyuki Tachibana,^c^ Taku Sato,^a^ Haruhisa Ohta,^a^ Motoaki Nakamura,^a^ Nobumasa Kato,^a^ Simon B. Eickhoff,^d,e^ Samuele Cortese,^f,g,h,I,j^ Yuta Y. Aoki^a*^

**Affiliations:**

a Medical Institute of Developmental Disabilities Research, Showa University, Tokyo, Japan.

b Department of Language Sciences, Graduate School of Humanities, Tokyo Metropolitan University, Tokyo, Japan.

c Division of Infant and Toddler Mental Health, Department of Psychosocial Medicine, National Center for Child Health and Development, Tokyo, Japan.

d Institute of Systems Neuroscience, Medical Faculty, Heinrich Heine University Düsseldorf, Düsseldorf, Germany

e Institute of Neuroscience and Medicine, Brain & Behaviour (INM-7), Research Centre Jülich, Jülich, Germany

f New York University Child Study Center, New York, NY, USA

g Center for Innovation in Mental Health, Academic Unit of Psychology, University of Southampton, UK

h Clinical and Experimental Sciences (CNS and Psychiatry), Faculty of Medicine, University of Southampton, UK

i Solent NHS Trust, Southampton, UK

j Division of Psychiatry and Applied Psychology, School of Medicine, University of Nottingham, Nottingham, UK

**Corresponding to**: Yuta Y. Aoki, Ph.D. MD.

Senior Assistant Professor

Medical Institute of Developmental Disabilities Research at Showa University

6-11-11 Kita-karasuyama, Setagaya-ku, Tokyo 157-8577, Japan


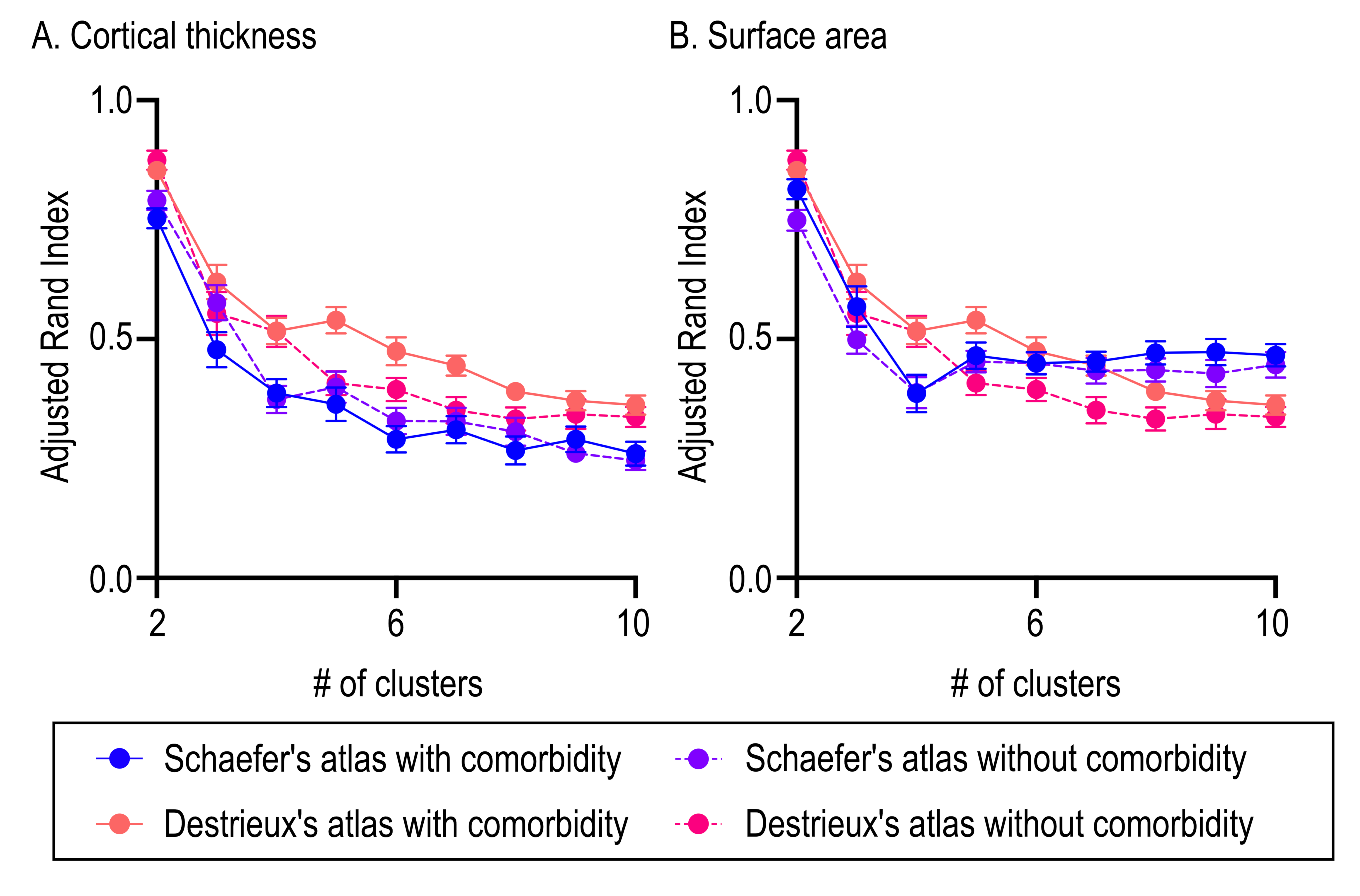


**Supplementary Figure 1. Plots of the number of clusters versus adjusted Rand index (ARI).** HYDRA identified that two subtypes within the group of developmental disorders was optimal for both cortical thickness (a) and surface area (b).


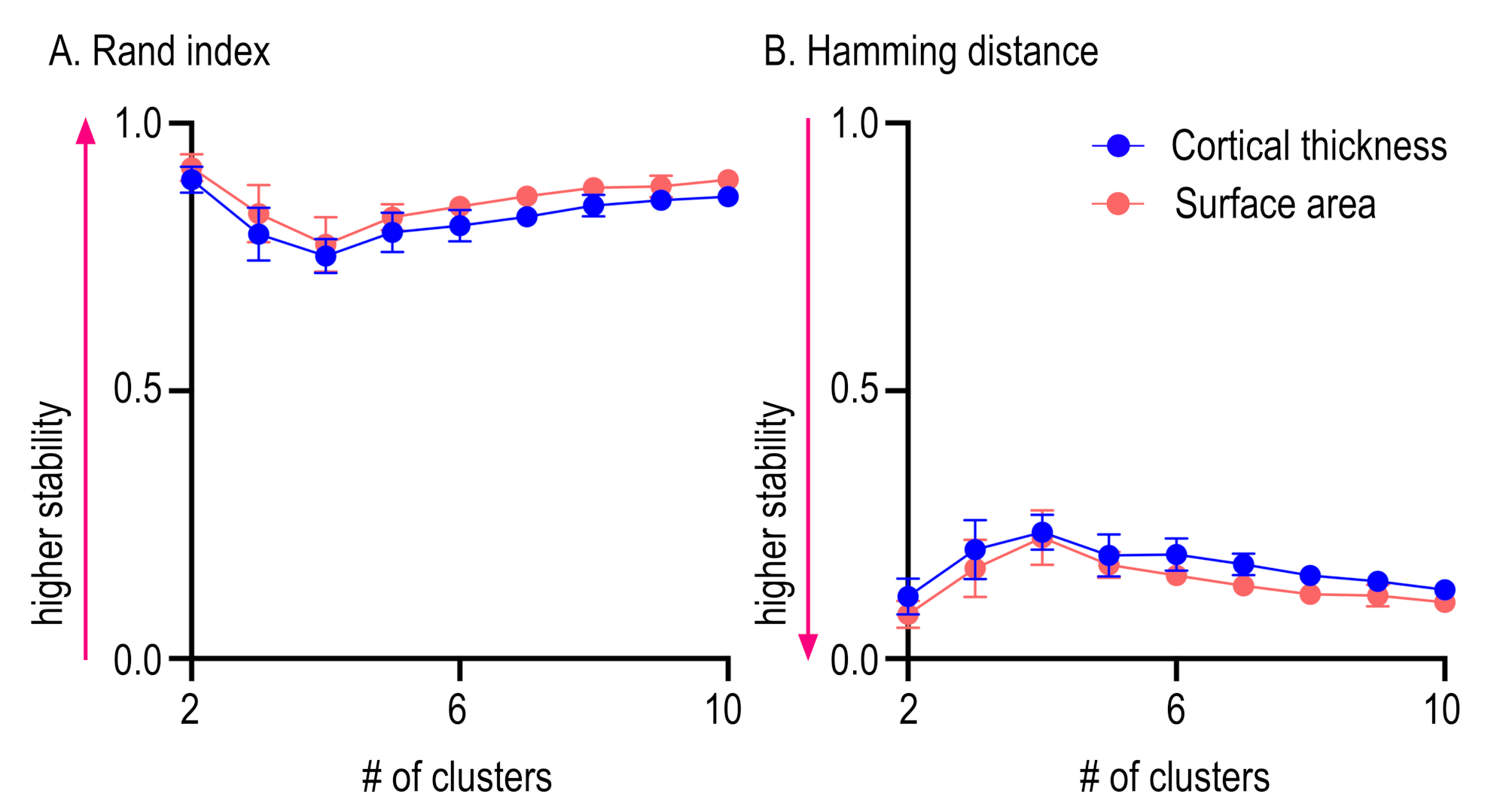


**Supplementary Figure 2.** Plots of the number of clusters versus Hamming distance (a) and Rand index (b). Both measures indicated that two subtypes were optimal for both cortical thickness (blue) and surface area (red).
